# Supplementary material for: Prevalence and incidence of hypertension in the global HIV-infected population: a systematic review and meta-analysis protocol
Source: BMJ Open. 2017 Oct 12;7(10):e016531. doi: 10.1136/bmjopen-2017-016531 (PMC5652496; doi:10.1136/bmjopen-2017-016531)
Supplement: Author's manuscript [file bmjopen-2017-016531.draft_revisions.pdf]

BMJ Open is committed to open peer review. As part of this commitment we make the peer review history of every article we publish publicly available.

When an article is published we post the peer reviewers' comments and the authors' responses online. We also post the versions of the paper that were used during peer review. These are the versions that the peer review comments apply to.

The versions of the paper that follow are the versions that were submitted during the peer review process. They are not the versions of record or the final published versions. They should not be cited or distributed as the published version of this manuscript.

BMJ Open is an open access journal and the full, final, typeset and author-corrected version of record of the manuscript is available on our site with no access controls, subscription charges or pay-per-view fees (http://bmjopen.bmj.com).

If you have any questions on BMJ Open's open peer review process please email info.bmjopen@bmj.com

BMJ Open

Prevalence and incidence of hypertension in the global HIV-infected population: a systematic review and meta-analysis protocol

Journal:	*BMJ Open*
Manuscript ID	bmjopen-2017-016531
Article Type:	Protocol
Date Submitted by the Author:	21-Feb-2017
Complete List of Authors:	Bigna, Jean Joel; Centre Pasteur of Cameroon, Department of Epidemiology and Public Health; University of Paris Sud XI, Faculty of Medicine<br>Tankeu, Aurel; University of Yaoundé 1, Department of Internal Medicine and Specialties, Faculty of Medicine and Biomedical Sciences<br>Kaze, Arnaud; Harvard Medical School, Brigham and Women's Hospital<br>Noubiap, Jean Jacques; Groote Schuur Hospital and University of Cape Town, Department of Medicine<br>Nansseu, Jobert Richie; University of Yaoundé 1, Department of Public Health, Faculty of Medicine and Biomedical Sciences; Ministry of Public Health, Department of Disease, Epidemics and Pandemics Control
**Primary Subject Heading**:	Cardiovascular medicine
Secondary Subject Heading:	Infectious diseases, Epidemiology, HIV/AIDS, Public health
Keywords:	HIV & AIDS < INFECTIOUS DISEASES, Hypertension < CARDIOLOGY, Epidemiology < INFECTIOUS DISEASES

SCHOLARONE™ Manuscripts

For peer review only

Prevalence and incidence of hypertension in the global HIV-infected population: a systematic review and meta-analysis protocol

Jean Joel R. Bigna[1, 2], Aurel T. Tankeu[3], Arnaud D. Kaze[4], Jean Jacques N. Noubiap[5], Jobert Richie N. Nansseu[6, 7]

[1-] Department of Epidemiology and Public Health, Centre Pasteur of Cameroon, Yaoundé, Cameroon

[2-] Faculty of Medicine, University of Paris Sud XI, Le Kremlin-Bicêtre, France

[3-] Department of Internal Medicine and Specialties, Faculty of Medicine and Biomedical Sciences, University of Yaoundé 1, Yaoundé, Cameroon

[4-] Brigham and Women's Hospital, Harvard Medical School, Boston, MA, USA

[5-] Department of Medicine, Groote Schuur Hospital and University of Cape Town, Cape Town, South Africa

[6-] Department of Public Health, Faculty of Medicine and Biomedical Sciences, University of Yaoundé 1, Yaoundé, Cameroon

[7-] Department of Disease, Epidemics and Pandemics Control, Ministry of Public Health, Yaoundé, Cameroon

* Correspondence to Dr Jean Joel R. Bigna, Department of Epidemiology and Public Health, Centre Pasteur of Cameroon, Yaoundé, Cameroon. Email: bignarimjj@yahoo.fr

E-mail addresses: JJRB: bignarimjj@yahoo.fr; ATT: aurelnet2014@yahoo.com; ADK: kazearnaud@yahoo.fr; JJNN: noubiapjj@yahoo.fr; JRNN: jobertrichie_nansseu@yahoo.fr

Keywords: hypertension, HIV, AIDS, epidemiology

For peer review only

Abstract

Introduction

Hypertension representing one of the most frequent cardiovascular risk factor, is thought to increase in individuals living with HIV as well as in general population but summarized global data on the topic are scare. We conduct this systematic review and meta-analysis to assess the prevalence/incidence of hypertension in the global HIV-infected population.

Methods and analysis

This review will include observational studies conducted among HIV infected people, which reported prevalence/incidence of hypertension or enough data for its appraisal. We will consider published and unpublished studies from January 1st, 1996 to February 28, 2017. Relevant records will be searched using PubMed/Medline and EMBASE. Reference lists of eligible papers and relevant review articles will be screened. Two investigators will independently screen, select studies, and extract data, with discrepancies resolved by consensus or by arbitration of a third investigator. Methodological quality of the included studies will be assessed using the scale developed by Hoy and colleagues. Funnel-plots and Egger's test will be used to determine publication bias. The study-specific estimates will be pooled through a random-effects meta-analysis model to obtain an overall summary estimate. To keep the effect of studies with extremely small or extremely large estimates on the overall estimate to a minimum, the variance of the study-specific prevalence/incidence will be stabilized with the Freeman-Tukey single arc-sine transformation. The heterogeneity will be evaluated by the χ^2 test on Cochrane's Q statistic. Results will be presented by geographic region, income and antiretroviral therapy status.

Ethic and Dissemination

For peer review only - http://bmjopen.bmj.com/site/about/guidelines.xhtml

This study is based on published data; therefore, ethical approval is not a requirement. The final report of this study in the form of a scientific paper will be published in peer-reviewed journals.

Review registration number

PROSPERO, CRD42016051684.

For peer review only

For peer review only - http://bmjopen.bmj.com/site/about/guidelines.xhtml

Strengths and limitations of this study

- To the best of our knowledge, this will be the first systematic review and meta-analysis which aims to estimate the global prevalence and incidence of hypertension in HIV infected people.
- We will use rigorous methodological and statistical procedures to derive accurate estimates.
- This review would be limited by the impossibility to disaggregate data between patients on antiretroviral therapy and those naïve of antiretroviral therapy.
- Another possible limitation could be the predominance of hospital-based studies limiting the generalizability of findings.

For peer review only

For peer review only - http://bmjopen.bmj.com/site/about/guidelines.xhtml

Introduction

The HIV pandemic is to-date one of the major global public health concern. Globally, there is 38.7 million people living with HIV in 2015 causing 35 million of deaths since the beginning of the epidemic.[1] Since the expanding used of highly active antiretroviral therapy (ART) in 1996, the natural history of HIV disease has been strikingly changed. This led to the increase of life expectancy in people living with HIV[1] and acceleration of epidemiological transition, from domination of the infectious diseases to non-communicable diseases.[2] The leading group of non-communicable diseases, cardiovascular diseases (CVDs), accounts for approximately 17 million deaths a year, nearly one third of the total worldwide. Of these, complications of hypertension account for more than half of deaths worldwide every year.[3 4]

Although the 38.7 million people living with HIV can now live longer because of ART, their life expectancy improvement can be associated with CVDs, including hypertension.[5-7] Given the recent World Health Organization (WHO) recommendations for initiating ART in all HIV-infected people regardless of CD4 count;[8 9] given the well established role of ART which damage the vascular endothelium responsible for the production of biological markers for blood pressure regulation;[5 6] it is important to focus on this high important modifiable cardiovascular risk factor in people living with HIV.

To date, although there are several original studies that report the prevalence and incidence of hypertension in people living with HIV, there is no systematic global evidence to the best of our knowledge. This review will be an opportunity to discuss epidemiological data across different WHO regions, income level, and socio-demographic profiles. We therefore plan to conduct this systematic review and meta-analysis to address this gap.

Review question

For peer review only - http://bmjopen.bmj.com/site/about/guidelines.xhtml

What is the prevalence and incidence of hypertension in the global HIV-infected population as documented in studies reported between January 1st, 1996 and February 28, 2017?

Objectives

To answer this review question, a systematic review and meta-analysis will be conducted. The objectives of which are to determine:

1) The prevalence and incidence of hypertension in the global HIV-infected ART-naive population,
2) The prevalence and incidence of hypertension in the global HIV-infected ART-experienced population.

Methods and analysis

Design

Centre for Reviews and Dissemination guidelines will be used for the methodology of this review.[10] This review is registered in the PROSPERO International Prospective Register of systematic reviews, registration number CRD42016051684.

Criteria for considering studies for this review

Inclusion criteria

1) Types of studies: observational studies (cross-sectional, case-control, or cohort studies).
2) Types of participants: HIV-infected ART-naïve people and HIV-infected ART-experienced people.

For peer review only - http://bmjopen.bmj.com/site/about/guidelines.xhtml

3) Type of outcome: hypertension defined as blood pressure ≥ 140/90 mmHg and/or treatment with anti-hypertensive medications.

4) Outcome measurement: prevalence/incidence or having enough data to compute these estimates.

5) Type of publication: published and unpublished studies reported from January 1st, 1996 to December 31st, 2016.

Exclusion criteria

1) Types of studies: case series, reviews, policy reports, commentaries and editorials.

2) Types of outcomes: studies on non-systemic and non-essential hypertension (intracranial hypertension, pulmonary hypertension).

3) Types of participants: studies in subgroups of participants selected on the basis of the presence of hypertension. Studies in which it will not be possible to differentiate HIV-infected ART-naive people and HIV-infected ART-experienced people. Studies conducted or in which it will not be possible to differentiate data of adults with those of children, adolescents, or pregnant women.

4) Outcome measurement: studies lacking primary data and/or explicit method description.

5) Duplicate reports: the most comprehensive and up-to-date version will be considered for this review.

Search strategy for identifying relevant studies

Bibliographic database search

For peer review only

A comprehensive and exhaustive search of PubMed/Medline and EMBASE will be performed to identify all relevant articles published on systemic hypertension among HIV-infected people between January1st, 1996 and February 28, 2017. A search strategy based on the combination of relevant terms will be conceived and applied. The following terms will be used for hypertension: “hypertension”, “high blood pressure”, “systolic hypertension”, “diastolic hypertension”, “blood pressure”, “anti-hypertensive”, and “hypertensive”. For HIV, we will use the terms “HIV”, “AIDS” and “antiretroviral”. The main search strategy conducted in PubMed/Medline is shown in Table 1. This search strategy will be adapted for searching EMBASE.

Searching other sources

A manual search which consists in scanning the reference lists of eligible papers and other relevant review articles will be conducted.

Selection of studies for inclusion in the review

Two investigators will independently identify articles and sequentially screen their titles and abstracts for eligibility. Full-texts of articles deemed potentially eligible will be retrieved. Further, these investigators will independently assess the full-text of each study for eligibility, and consensually retain studies to be included. Disagreements when existing will be solved by a third investigator. A screening guide will be used to ensure that the selection criteria are reliably applied by all investigators. Selection of studies will be managed using EndNote X7.

Data extraction and management

For peer review only - http://bmjopen.bmj.com/site/about/guidelines.xhtml

Two investigators will independently extract data pertaining to:

1) Author details: name of first author and publication year.

2) Study characteristics: country, WHO region (African Region, Regions of Americas, South-East Asia Region, European Region, Eastern Mediterranean Region, and Western Pacific Region), World Bank classification by income level at the time of data collection (low-income, lower-middle-income, upper-middle income, high-income countries), study design (cross sectional, case-control, cohort), setting (urban and/or rural), sampling method (random vs non-random), data collection period, response rate, timing of data collection (prospectively vs retrospectively), and methodological quality of the study.

3) Participants' characteristics: selection criteria, age (mean, median, range), gender (proportion of male), HIV related data (time since diagnosis, severity of the disease, ART regimens and duration of treatment).

4) Hypertension characteristics: diagnostic criteria used, prevalence, number of participants tested and diagnosed with hypertension overall and by subgroup of interest like level of urbanization, gender and ART regimens and status.

Where only primary data (sample size and number of outcomes) will be provided, these data will be used to calculate the prevalence or incidence estimates. Data will be extracted using a preconceived and standardized data abstraction form. Disagreements between investigators will be reconciled through discussion and consensus, or arbitration by a third investigator whenever necessary. In case of multinational studies, the results will be separated to show the estimate within individual countries. When it will not be possible to disaggregate the data by country, the study will be presented as one and the countries in which the study was done will be shown.

For peer review only - http://bmjopen.bmj.com/site/about/guidelines.xhtml

Appraisal of the methodological quality of included studies and risk of bias

Included studies will be evaluated for methodological quality in terms of internal validity, external validity, response rate, and generalizability of study results. We will adapt the 10-item rating scale developed Hoy et al (Table 2).[11] Each item will be assigned a score of 1 (Yes) or 0 (No), and score will be summed across items to generate an overall quality score. The total score will range from 0 to 10 with the overall score categorized as follows: 8-10: “low risk of bias”, 5-7: “moderate risk”, and 0-4: “high risk”. We intend to present risk of bias and quality scores in a table.

Data synthesis including assessment of heterogeneity

Data will be analyzed using Stata software (Stata Corp V.14, Texas, USA). Unadjusted prevalence/incidence and standard errors of hypertension will be recalculated based on the information of crude numerators and denominators provided by individual studies. To keep the effect of studies with extremely small or extremely large prevalence estimates on the overall estimate to a minimum, the variance of the study-specific prevalence/incidence will be stabilized with the Freeman-Tukey single arc-sine transformation before pooling the data with the random-effects meta-analysis model.[12] Heterogeneity will be evaluated by the Chi-squared test on Cochrane’s Q statistic,[13] which will be quantified by I-squared values, assuming that I-squared values of 25%, 50% and 75% being representative of low, medium and high heterogeneity respectively.[14] When substantial heterogeneity will be detected, we will perform a subgroup analysis to investigate the possible sources of heterogeneity using the following grouping variables: age group, sex, study setting (rural vs urban), geographical

For peer review only - http://bmjopen.bmj.com/site/about/guidelines.xhtml

area, country income level, sampling method, timing of data collection, ART regimens and status, hypertension diagnosis criteria, and study methodological quality. We will assess inter-rater agreement between investigators for study inclusion, data extraction, and methodological quality assessment using Kappa Cohen's coefficient.[15] If the included studies differ significantly in design, settings, outcome measures or otherwise, a narrative format will be used to summarize data of studies.

Assessment of reporting biases

Symmetry of funnel plots and Egger's test will be done to assess the presence of publication and selective reporting bias.[16] A p-value < 0.10 will be considered indicative of statistically significant publication bias. In addition, we will perform a trim-and-fill adjusted analysis. This help to perform the Duval and Tweedie nonparametric "trim and fill" method of accounting for publication bias in meta-analysis and estimates the number and outcomes of missing studies, and adjusts the meta-analysis to incorporate the theoretical missing studies.[17]

Reporting of this review

Results will be presented by geographic region, country income level and antiretroviral therapy status. The guidelines for meta-analyses and systematic reviews of Preferred Reporting Items for Systematic Review and Meta-Analysis (PRISMA) will serve as the template for reporting the present review.[18] For the present protocol, the PRISMA for Protocol was used for the reporting (see online supplementary appendix S1).[19]

For peer review only - http://bmjopen.bmj.com/site/about/guidelines.xhtml

Potential amendments

We do not intend to make any amendments to the protocol, to avoid the possibility of outcome reporting bias. However, any amendments during review process will be reported transparently.

Conclusions

Reduction of mortality and morbidity gained in HIV infected individuals with the widespread use of HAART has been accompanied by an increasing prevalence of CVD in this population. [5-7] Hypertension which as far the most frequent and worrying cardiovascular risk factor is increasing among HIV infected patients as in general population. In this context, we plan to conduct this review with the aim of estimating the burden of this condition in this population. We hope that this review will serve to draw attention and raise awareness on this growing concern.

Ethics and dissemination

The current study is based on published data and as such, ethical approval is not a requirement. The final report of the systematic review in the form of a scientific paper will be published in peer-reviewed journals. Findings will further be presented at conferences and submitted to relevant health authorities. We also plan to update the review in the future to monitor changes and guide health service and policy solutions.

Competing interests

For peer review only

For peer review only - http://bmjopen.bmj.com/site/about/guidelines.xhtml

None.

Contributors

Conception and design of the protocol: ATT and JJRB. First draft: ATT and JJRB. Critical revision of the manuscript for methodological and intellectual content: JRNN, ADK, and JJNN. Guarantor of the review: JJRB. All authors approved the final version of the submitted manuscript.

Funding

This research received no specific grant from any funding agency in the public, commercial or not-for-profit sectors.

Data sharing statement

No additional data are available.

References

1. UNAIDS. Fact sheet 2016 Global statistics-2015. 2016. http://www.unaids.org/en/resources/fact-sheet.

2. Kuate Defo B. Demographic, epidemiological, and health transitions: are they relevant to population health patterns in Africa? Global health action 2014;**7**:22443.

3. World Health Organization. A global brief on hypertension. 2013. http://apps.who.int/iris/bitstream/10665/79059/1/WHO_DCO_WHD_2013.2_eng.pd.

4. Lim SS, Vos T, Flaxman AD, et al. A comparative risk assessment of burden of disease and injury attributable to 67 risk factors and risk factor clusters in 21 regions, 1990-2010: a systematic analysis for the Global Burden of Disease Study 2010. Lancet 2012;**380**(9859):2224-60.

5. Dillon DG, et al. Association of HIV and ART with cardiometabolic traits in sub-Saharan Africa: a systematic review and meta-analysis. Int J Epidemiol 2016.

6. Nduka CU, Stranges S, Sarki AM, et al. Evidence of increased blood pressure and hypertension risk among people living with HIV on antiretroviral therapy: a systematic review with meta-analysis. Journal of human hypertension 2016;**30**(6):355-62.

For peer review only - http://bmjopen.bmj.com/site/about/guidelines.xhtml

For peer review only

7. Nansseu JR, Bigna JJ. Antiretroviral therapy related adverse effects: Can sub-Saharan Africa cope with the new "test and treat" policy of the World Health Organization? Infectious diseases of poverty 2017;**6**(1):24.

8. World health Organization. Consolidated guidelines on the use of antiretroviral drugs for treating and preventing HIV infection - Recommendations for a public health approach - Second edition. 2016. http://www.who.int/hiv/pub/arv/arv-2016/en/.

9. Bigna JJ, Plottel CS, Koulla-Shiro S. Challenges in initiating antiretroviral therapy for all HIV-infected people regardless of CD4 cell count. Infectious diseases of poverty 2016;**5**:85.

10. Centers for Reviews and Dissemination. CRD's guidance for undertaking reviews in healthcare: Centers for Reviews and Dissemination, 2009.

11. Hoy D, Brooks P, Woolf A, et al. Assessing risk of bias in prevalence studies: modification of an existing tool and evidence of interrater agreement. Journal of clinical epidemiology 2012;**65**(9):934-9.

12. Barendregt JJ, Doi SA, Lee YY, et al. Meta-analysis of prevalence. J Epidemiol Community Health 2013;**67**(11):974-8.

13. Cochran GW. The Combination of Estimates from Different Experiments. Biometrics 1954;**10**(1):101-29.

14. Higgins JP, Thompson SG. Quantifying heterogeneity in a meta-analysis. Statistics in medicine 2002;**21**(11):1539-58.

15. Viera AJ, Garrett JM. Understanding interobserver agreement: the kappa statistic. Fam Med 2005;**37**(5):360-3.

16. Egger M, Davey Smith G, Schneider M, et al. Bias in meta-analysis detected by a simple, graphical test. BMJ 1997;**315**(7109):629-34.

17. Duval S, Tweedie R. Trim and fill: A simple funnel-plot-based method of testing and adjusting for publication bias in meta-analysis. Biometrics 2000;**56**(2):455-63.

18. Knobloch K, Yoon U, Vogt PM. Preferred reporting items for systematic reviews and meta-analyses (PRISMA) statement and publication bias. Journal of cranio-maxillo-facial surgery : official publication of the European Association for Cranio-Maxillo-Facial Surgery 2011;**39**(2):91-2.

19. Moher D, Shamseer L, Clarke M, et al. Preferred reporting items for systematic review and meta-analysis protocols (PRISMA-P) 2015 statement. Systematic reviews 2015;**4**:1.

For peer review only

Table 1. Search strategy in PubMed

Search	Search terms
#1	"HIV" OR "AIDS" OR "antiretroviral therapy" OR "antiretroviral treatment" OR "highly active antiretroviral therapy" OR "highly active ART"
#2	"Hypertension" OR "high blood pressure" OR "systolic hypertension" OR "diastolic hypertension" OR "anti-hypertensive" OR "blood pressure" OR "hypertensive"
#3	#1 AND #2
#4	#3 Limits: from 1996/01/01 to 2017/02/28

For peer review only - http://bmjopen.bmj.com/site/about/guidelines.xhtml

Table 2. Risk of bias assessment tool

**Risk of Bias Item***
External Validity
1. Was the study target population a close representation of the HIV population in relation to relevant variables?
2. Was the sampling frame a true or close representation of the target population?
3. Was some form of random selection used to select the sample, OR, was a census undertaken?
4. Was the likelihood of non-participation bias minimal?
Internal Validity
5. Were data collected directly from the subjects (as opposed to medical records)?
6. Were acceptable case definition of hypertension used?
7. Was a reliable and accepted diagnosis method for hypertension utilized?
8. Was the same mode of data collection used for all subjects?
9. Was the length of the shortest prevalence period for the parameter of interest appropriate?
10. Were the numerator(s) and denominator(s) for the calculation of the prevalence/incidence of hypertension appropriate?

*Adapted from the Risk of Bias Tool for Prevalence Studies developed by Hoy et al

For peer review only

For peer review only - http://bmjopen.bmj.com/site/about/guidelines.xhtml

PRISMA-P (Preferred Reporting Items for Systematic review and Meta-Analysis Protocols) 2015 checklist: recommended items to address in a systematic review protocol

Section and topic	Item No	Checklist item	
**ADMINISTRATIVE INFORMATION**			
Title:			
Identification	1a	Identify the report as a protocol of a systematic review	1
Update	1b	If the protocol is for an update of a previous systematic review, identify as such	-
Registration	2	If registered, provide the name of the registry (such as PROSPERO) and registration number	3
Authors:			
Contact	3a	Provide name, institutional affiliation, e-mail address of all protocol authors; provide physical mailing address of corresponding author	1
Contributions	3b	Describe contributions of protocol authors and identify the guarantor of the review	13
Amendments	4	If the protocol represents an amendment of a previously completed or published protocol, identify as such and list changes; otherwise, state plan for documenting important protocol amendments	-
Support:			
Sources	5a	Indicate sources of financial or other support for the review	13
Sponsor	5b	Provide name for the review funder and/or sponsor	-
Role of sponsor or funder	5c	Describe roles of funder(s), sponsor(s), and/or institution(s), if any, in developing the protocol	-
**INTRODUCTION**			
Rationale	6	Describe the rationale for the review in the context of what is already known	5
Objectives	7	Provide an explicit statement of the question(s) the review will address with reference to participants, interventions, comparators, and outcomes (PICO)	6
**METHODS**			
Eligibility criteria	8	Specify the study characteristics (such as PICO, study design, setting, time frame) and report characteristics (such as years considered, language, publication status) to be used as criteria for eligibility for the review	6-7
Information sources	9	Describe all intended information sources (such as electronic databases, contact with study authors, trial registers or other grey literature sources) with planned dates of coverage	7-8
Search strategy	10	Present draft of search strategy to be used for at least one electronic database, including planned limits, such that it could be repeated	7-8
Study records:			
Data management	11a	Describe the mechanism(s) that will be used to manage records and data throughout the review	8-9
Selection	11b	State the process that will be used for selecting studies (such as two independent reviewers) through each phase of the review	7

For peer review only - http://bmjopen.bmj.com/site/about/guidelines.xhtml

process		(that is, screening, eligibility and inclusion in meta-analysis)	
Data collection process	11c	Describe planned method of extracting data from reports (such as piloting forms, done independently, in duplicate), any processes for obtaining and confirming data from investigators	8-9
Data items	12	List and define all variables for which data will be sought (such as PICO items, funding sources), any pre-planned data assumptions and simplifications	8-9
Outcomes and prioritization	13	List and define all outcomes for which data will be sought, including prioritization of main and additional outcomes, with rationale	8-9
Risk of bias in individual studies	14	Describe anticipated methods for assessing risk of bias of individual studies, including whether this will be done at the outcome or study level, or both; state how this information will be used in data synthesis	10
Data synthesis	15a	Describe criteria under which study data will be quantitatively synthesised	10-11
	15b	If data are appropriate for quantitative synthesis, describe planned summary measures, methods of handling data and methods of combining data from studies, including any planned exploration of consistency (such as I^2, Kendall's τ)	10-11
	15c	Describe any proposed additional analyses (such as sensitivity or subgroup analyses, meta-regression)	10-11
	15d	If quantitative synthesis is not appropriate, describe the type of summary planned	10-11
Meta-bias(es)	16	Specify any planned assessment of meta-bias(es) (such as publication bias across studies, selective reporting within studies)	10-11
Confidence in cumulative evidence	17	Describe how the strength of the body of evidence will be assessed (such as GRADE)	10-11

For peer review only

BMJ Open

Prevalence and incidence of hypertension in the global HIV-infected population: a systematic review and meta-analysis protocol

Journal:	*BMJ Open*
Manuscript ID	bmjopen-2017-016531.R1
Article Type:	Protocol
Date Submitted by the Author:	16-Jun-2017

Complete List of Authors:	Bigna, Jean Joel; Centre Pasteur of Cameroon, Department of Epidemiology and Public Health; University of Paris Sud XI, Faculty of Medicine<br>Tankeu, Aurel; University of Yaoundé 1, Department of Internal Medicine and Specialties, Faculty of Medicine and Biomedical Sciences<br>Kaze, Arnaud; Harvard Medical School, Brigham and Women's Hospital<br>Noubiap, Jean Jacques; Groote Schuur Hospital and University of Cape Town, Department of Medicine<br>Nansseu, Jobert Richie; University of Yaoundé 1, Department of Public Health, Faculty of Medicine and Biomedical Sciences; Ministry of Public Health, Department of Disease, Epidemics and Pandemics Control

**Primary Subject Heading**:	Cardiovascular medicine
Secondary Subject Heading:	Infectious diseases, Epidemiology, HIV/AIDS, Public health
Keywords:	HIV & AIDS < INFECTIOUS DISEASES, Hypertension < CARDIOLOGY, Epidemiology < INFECTIOUS DISEASES

SCHOLARONE™ Manuscripts

Prevalence and incidence of hypertension in the global HIV-infected population: a systematic review and meta-analysis protocol

Jean Joel Bigna[1, 2], Aurel T. Tankeu[3], Arnaud D. Kaze[4], Jean Jacques Noubiap[5], Jobert Richie Nansseu[6, 7]

[1-] Department of Epidemiology and Public Health, Centre Pasteur of Cameroon, Yaoundé, Cameroon

[2-] Faculty of Medicine, University of Paris Sud XI, Le Kremlin-Bicêtre, France

[3-] Department of Internal Medicine and Specialties, Faculty of Medicine and Biomedical Sciences, University of Yaoundé 1, Yaoundé, Cameroon

[4-] Brigham and Women's Hospital, Harvard Medical School, Boston, MA, USA

[5-] Department of Medicine, Groote Schuur Hospital and University of Cape Town, Cape Town, South Africa

[6-] Department of Public Health, Faculty of Medicine and Biomedical Sciences, University of Yaoundé 1, Yaoundé, Cameroon

[7-] Department of Disease, Epidemics and Pandemics Control, Ministry of Public Health, Yaoundé, Cameroon

* Correspondence to Dr Jean Joel Bigna, Department of Epidemiology and Public Health, Centre Pasteur of Cameroon, Yaoundé, Cameroon. Email: bignarimjj@yahoo.fr

E-mail addresses: JJB: bignarimjj@yahoo.fr; ATT: aurelnet2014@yahoo.com; ADK: kazearnaud@yahoo.fr; JJN: noubiapjj@yahoo.fr; JRN: jobertrichie_nansseu@yahoo.fr

Keywords: hypertension, HIV, AIDS, epidemiology

For peer review only

Abstract

Introduction

Hypertension representing one of the most frequent cardiovascular risk factor, is thought to increase in individuals living with HIV as well as in general population but summarized global data on the topic are scare. We conduct this systematic review and meta-analysis to assess the prevalence/incidence of hypertension in the global HIV-infected population.

Methods and analysis

This review will include observational studies conducted among HIV infected people, which reported prevalence/incidence of hypertension or enough data for its appraisal. We will consider published and unpublished studies from January 1, 2007 to May 31, 2017. Relevant records will be searched using PubMed/Medline, Global Index Medicus, Web of Science, and EMBASE. Reference lists of eligible papers and relevant review articles will be screened. Two investigators will independently screen, select studies, and extract data, with discrepancies resolved by consensus or by arbitration of a third investigator. Methodological quality of the included studies will be assessed using the scale developed by Hoy and colleagues. Funnel-plots and Egger's test will be used to determine publication bias. The study-specific estimates will be pooled through a random-effects meta-analysis model to obtain an overall summary estimate. To keep the effect of studies with extremely small or extremely large estimates on the overall estimate to a minimum, the variance of the study-specific prevalence/incidence will be stabilized with the Freeman-Tukey single arc-sine transformation. The heterogeneity will be evaluated by the χ^2 test on Cochrane's Q statistic. Results will be presented by geographic region, income and antiretroviral therapy status.

Ethic and Dissemination

For peer review only - http://bmjopen.bmj.com/site/about/guidelines.xhtml

This study is based on published data; therefore, ethical approval is not a requirement. The final report of this study in the form of a scientific paper will be published in peer-reviewed journals.

Review registration number

PROSPERO, CRD42016051684.

For peer review only - http://bmjopen.bmj.com/site/about/guidelines.xhtml

Strengths and limitations of this study

- To the best of our knowledge, this will be the first systematic review and meta-analysis which aims to estimate the global prevalence and incidence of hypertension in HIV infected people.
- We will use rigorous methodological and statistical procedures to derive accurate estimates.
- This review would be limited by the impossibility to disaggregate data between patients on antiretroviral therapy and those naïve of antiretroviral therapy.
- Another possible limitation could be the predominance of hospital-based studies limiting the generalizability of findings.

For peer review only

Introduction

The HIV pandemic is to-date one of the major global public health concern. Globally, there were 38.7 million people living with HIV in 2015 causing 35 million of deaths since the beginning of the epidemic.[1] Since the expanding use of highly active antiretroviral therapy (ART) in 1996, the natural history of HIV disease has been strikingly changed. This led to the increase of life expectancy in people living with HIV[1] and acceleration of epidemiological transition, from domination of the infectious diseases to non-communicable diseases.[2] The leading group of non-communicable diseases, cardiovascular diseases (CVDs), accounts for approximately 17 million deaths a year, nearly one third of the total worldwide. Of these, complications of hypertension account for more than half of deaths worldwide every year.[3 4]

Although the 38.7 million people living with HIV can now live longer because of ART, the risk of CVD including hypertension increases with aging and other factors such as diet, physical inactivity, smoking, and hyperuricemia.[5-8] This is due to both HIV and ART. Indeed, several HIV related factors have been mentioned to cause vascular dysfunction by HIV itself including endothelial dysfunction, endothelial cells activation through viral proteins action, activation of macrophages responsible of accelerated atheroma formation, HIV-associated lipid disorders, proinflammatory and prothrombotic state, and direct HIV infection of endothelial cells and vascular smooth muscle cells.[9 10] In addition to HIV itself, ART have been incriminated to increase the oxidative stress in endothelial cells, to favor adhesion of mononuclear on vascular endothelium and insulin resistance, to increase accumulation of fatty acids and lipids on vessel wall, and to favor persistent immunity activity and inflammation.[10] Autonomic neuropathy and vasculitis were also evocated as potential mechanism for the occurrence of hypertension in HIV people by a secondary pathogenic pathway.[9] Given the recent World Health Organization (WHO) recommendations for initiating ART in all HIV-infected people regardless of CD4 count;[11 12] people living with

For peer review only - http://bmjopen.bmj.com/site/about/guidelines.xhtml

HIV will face the double burden of HIV infection and HIV and ART induced CVDs including hypertension. One can also wonder whether countries with weak health system are ready to face these challenges.[7 12] This is why it is important to know what is the burden of CVDs in people living with HIV?

To date, although there are several original studies that report the prevalence and incidence of hypertension in people living with HIV, there is no systematic global evidence to the best of our knowledge. This review will be an opportunity to discuss epidemiological data across different WHO regions, income level, and socio-demographic profiles. We therefore plan to conduct this systematic review and meta-analysis to address this gap.

Review question

What is the prevalence and incidence of hypertension in the global HIV-infected population as documented in studies reported between January 1st, 1996 and February 28, 2017?

Objectives

To answer this review question, a systematic review and meta-analysis will be conducted. The objectives of which are to determine:

1) The prevalence and incidence of hypertension in the global HIV-infected ART-naive population,
2) The prevalence and incidence of hypertension in the global HIV-infected ART-experienced population.

Methods and analysis

For peer review only - http://bmjopen.bmj.com/site/about/guidelines.xhtml

Design

Centre for Reviews and Dissemination guidelines will be used for the methodology of this review.[13] This review is registered in the PROSPERO International Prospective Register of systematic reviews, registration number CRD42016051684.

Criteria for considering studies for this review

Inclusion criteria

1) Types of studies: observational studies (cross-sectional, case-control, or cohort studies).
2) Types of participants: HIV-infected ART-naïve people and HIV-infected ART-experienced people.
3) Type of outcome: hypertension defined as office blood pressure ≥ 140/90 mmHg and/or treatment with anti-hypertensive medications.
4) Outcome measurement: prevalence/incidence or having enough data to compute these estimates.
5) Type of publication: published and unpublished studies reported from January 1, 2007 to May 31, 2017.

Exclusion criteria

1) Types of studies: case series, reviews, policy reports, commentaries and editorials.
2) Types of outcomes: studies on non-systemic and non-essential hypertension (intracranial hypertension, pulmonary hypertension).

For peer review only - http://bmjopen.bmj.com/site/about/guidelines.xhtml

3) Types of participants: studies in subgroups of participants selected on the basis of the presence of hypertension. Studies in which it will not be possible to differentiate HIV-infected ART-naive people and HIV-infected ART-experienced people. Studies conducted or in which it will not be possible to differentiate data of adults with those of children, adolescents, or pregnant women.

4) Outcome measurement: studies lacking primary data and/or explicit method description.

5) Duplicate reports: the most comprehensive and up-to-date version will be considered for this review.

Search strategy for identifying relevant studies

Bibliographic database search

A comprehensive and exhaustive search of PubMed/Medline, Global Index Medicus, Web of Science, and EMBASE will be performed to identify all relevant articles published on systemic hypertension among HIV-infected people between January 1, 2007 and May 31, 2017. A search strategy based on the combination of relevant terms will be conceived and applied. The following terms will be used for hypertension: “hypertension”, “high blood pressure”, “systolic hypertension”, “diastolic hypertension”, “blood pressure”, “anti-hypertensive”, and “hypertensive”. For HIV, we will use the terms “HIV”, “AIDS” and “antiretroviral”. The main search strategy conducted in PubMed/Medline is shown in Table 1. This search strategy will be adapted for searching other database.

Searching other sources

For peer review only - http://bmjopen.bmj.com/site/about/guidelines.xhtml

For peer review only

A manual search which consists in scanning the reference lists of eligible papers and other relevant review articles will be conducted.

Selection of studies for inclusion in the review

Two investigators will independently identify articles and sequentially screen their titles and abstracts for eligibility. Full-texts of articles deemed potentially eligible will be retrieved. Further, these investigators will independently assess the full-text of each study for eligibility, and consensually retain studies to be included. Disagreements when existing will be solved by a third investigator. A screening guide will be used to ensure that the selection criteria are reliably applied by all investigators. Selection of studies will be managed using EndNote X7.

Data extraction and management

Two investigators will independently extract data pertaining to:

1) Author details: name of first author and publication year.

2) Study characteristics: country, WHO region (African Region, Regions of Americas, South-East Asia Region, European Region, Eastern Mediterranean Region, and Western Pacific Region), World Bank classification by income level at the time of data collection (low-income, lower-middle-income, upper-middle income, high-income countries), study design (cross sectional, case-control, cohort), setting (urban and/or rural), sampling method (random vs non-random), data collection period, response rate, timing of data collection (prospectively vs retrospectively), and methodological quality of the study.

For peer review only - http://bmjopen.bmj.com/site/about/guidelines.xhtml

3) Participants' characteristics: selection criteria, age (mean, median, range), gender (proportion of male), HIV related data (time since diagnosis, severity of the disease, ART regimens and duration of treatment).

4) Hypertension characteristics: diagnostic criteria used, prevalence, number of participants tested and diagnosed with hypertension overall and by subgroup of interest like level of urbanization, gender and ART regimens and status.

Where only primary data (sample size and number of outcomes) will be provided, these data will be used to calculate the prevalence or incidence estimates. Data will be extracted using a preconceived and standardized data abstraction form. Disagreements between investigators will be reconciled through discussion and consensus, or arbitration by a third investigator whenever necessary. In case of multinational studies, the results will be separated to show the estimate within individual countries. When it will not be possible to disaggregate the data by country, the study will be presented as one and the countries in which the study was done will be shown.

Appraisal of the methodological quality of included studies and risk of bias

Included studies will be evaluated for methodological quality in terms of internal validity, external validity, response rate, and generalizability of study results. We will adapt the 10-item rating scale developed Hoy et al (Table 2).[14] Each item will be assigned a score of 1 (Yes) or 0 (No), and score will be summed across items to generate an overall quality score. The total score will range from 0 to 10 with the overall score categorized as follows: 8-10: "low risk of bias", 5-7: "moderate risk", and 0-4: "high risk". We intend to present risk of bias and quality scores in a table.

For peer review only - http://bmjopen.bmj.com/site/about/guidelines.xhtml

Data synthesis including assessment of heterogeneity

Data will be analyzed using Stata software (Stata Corp V.14, Texas, USA). Unadjusted prevalence/incidence and standard errors of hypertension will be recalculated based on the information of crude numerators and denominators provided by individual studies. To keep the effect of studies with extremely small or extremely large prevalence estimates on the overall estimate to a minimum, the variance of the study-specific prevalence/incidence will be stabilized with the Freeman-Tukey single arc-sine transformation before pooling the data with the random-effects meta-analysis model.[15] Heterogeneity will be evaluated by the Chi-squared test on Cochrane's Q statistic,[16] which will be quantified by I-squared values, assuming that I-squared values of 25%, 50% and 75% being representative of low, medium and high heterogeneity respectively.[17] When substantial heterogeneity will be detected, we will perform a subgroup and metaregression analyses to investigate the possible sources of heterogeneity using the following grouping variables: age group, sex, study setting (rural vs urban), geographical area, country income level, sampling method, timing of data collection, ART regimens and status, hypertension diagnosis criteria, and study methodological quality. We will assess inter-rater agreement between investigators for study inclusion, data extraction, and methodological quality assessment using Kappa Cohen's coefficient.[18] If the included studies differ significantly in design, settings, outcome measures or otherwise, a narrative format will be used to summarize data of studies.

Assessment of reporting biases

Symmetry of funnel plots and Egger's test will be done to assess the presence of publication and selective reporting bias.[19] A p-value < 0.10 will be considered indicative of statistically significant publication bias. In addition, we will perform a trim-and-fill adjusted analysis.

For peer review only - http://bmjopen.bmj.com/site/about/guidelines.xhtml

This help to perform the Duval and Tweedie nonparametric "trim and fill" method of accounting for publication bias in meta-analysis and estimates the number and outcomes of missing studies, and adjusts the meta-analysis to incorporate the theoretical missing studies.[20]

Reporting of this review

Results will be presented by geographic region, country income level and antiretroviral therapy status. The guidelines for meta-analyses and systematic reviews of Preferred Reporting Items for Systematic Review and Meta-Analysis (PRISMA) will serve as the template for reporting the present review.[21] For the present protocol, the PRISMA for Protocol was used for the reporting (see online supplementary appendix S1).[22]

Potential amendments

We do not intend to make any amendments to the protocol, to avoid the possibility of outcome reporting bias. However, any amendments during review process will be reported transparently.

Conclusions

Reduction of mortality and morbidity gained in HIV infected individuals with the widespread use of HAART has been accompanied by an increasing prevalence of CVD in this population. [5-7] Hypertension which as far the most frequent and worrying cardiovascular risk factor is increasing among HIV infected patients as in general population. In this context, we

For peer review only - http://bmjopen.bmj.com/site/about/guidelines.xhtml

plan to conduct this review with the aim of estimating the burden of this condition in this population. We hope that this review will serve to draw attention and raise awareness on this growing concern.

Ethics and dissemination

The current study is based on published data and as such, ethical approval is not a requirement. The final report of the systematic review in the form of a scientific paper will be published in peer-reviewed journals. Findings will further be presented at conferences and submitted to relevant health authorities. We also plan to update the review in the future to monitor changes and guide health service and policy solutions.

Competing interests

None.

Contributors

Conception and design of the protocol: ATT and JJRB. First draft: ATT and JJRB. Critical revision of the manuscript for methodological and intellectual content: JRNN, ADK, and JJNN. Guarantor of the review: JJRB. All authors approved the final version of the submitted manuscript.

Funding

This research received no specific grant from any funding agency in the public, commercial or not-for-profit sectors.

Data sharing statement

For peer review only - http://bmjopen.bmj.com/site/about/guidelines.xhtml

No additional data are available.

References

1. UNAIDS. Fact sheet 2016 Global statistics-2015. Secondary Fact sheet 2016 Global statistics-2015 2016 2016. http://www.unaids.org/en/resources/fact-sheet.

2. Kuate Defo B. Demographic, epidemiological, and health transitions: are they relevant to population health patterns in Africa? Global health action 2014;**7**:22443.

3. World Health Organization. A global brief on hypertension. Secondary A global brief on hypertension 2013. http://apps.who.int/iris/bitstream/10665/79059/1/WHO_DCO_WHD_2013.2_eng.pd.

4. Lim SS, Vos T, Flaxman AD, et al. A comparative risk assessment of burden of disease and injury attributable to 67 risk factors and risk factor clusters in 21 regions, 1990-2010: a systematic analysis for the Global Burden of Disease Study 2010. Lancet 2012;**380**(9859):2224-60.

5. Dillon DG, et al. Association of HIV and ART with cardiometabolic traits in sub-Saharan Africa: a systematic review and meta-analysis. Int J Epidemiol 2016.

6. Nduka CU, Stranges S, Sarki AM, et al. Evidence of increased blood pressure and hypertension risk among people living with HIV on antiretroviral therapy: a systematic review with meta-analysis. Journal of human hypertension 2016;**30**(6):355-62.

7. Nansseu JR, Bigna JJ. Antiretroviral therapy related adverse effects: Can sub-Saharan Africa cope with the new "test and treat" policy of the World Health Organization? Infectious diseases of poverty 2017;**6**(1):24.

8. Ances BM, Bhatt A, Vaida F, et al. Role of metabolic syndrome components in human immunodeficiency virus-associated stroke. Journal of neurovirology 2009;**15**(3):249-56.

9. Calo LA, Caielli P, Maiolino G, et al. Arterial hypertension and cardiovascular risk in HIV-infected patients. Journal of cardiovascular medicine (Hagerstown, Md) 2013;**14**(8):553-8.

10. Dube MP, Lipshultz SE, Fichtenbaum CJ, et al. Effects of HIV infection and antiretroviral therapy on the heart and vasculature. Circulation 2008;**118**(2):e36-40.

11. World health Organization. Consolidated guidelines on the use of antiretroviral drugs for treating and preventing HIV infection - Recommendations for a public health approach - Second edition. Secondary Consolidated guidelines on the use of antiretroviral drugs for treating and preventing HIV infection - Recommendations for a public health approach - Second edition June 2016 2016. http://www.who.int/hiv/pub/arv/arv-2016/en/.

For peer review only - http://bmjopen.bmj.com/site/about/guidelines.xhtml

12. Bigna JJ, Plottel CS, Koulla-Shiro S. Challenges in initiating antiretroviral therapy for all HIV-infected people regardless of CD4 cell count. Infectious diseases of poverty 2016;**5**:85.

13. Centers for Reviews and Dissemination. CRD's guidance for undertaking reviews in healthcare: Centers for Reviews and Dissemination, 2009.

14. Hoy D, Brooks P, Woolf A, et al. Assessing risk of bias in prevalence studies: modification of an existing tool and evidence of interrater agreement. Journal of clinical epidemiology 2012;**65**(9):934-9.

15. Barendregt JJ, Doi SA, Lee YY, et al. Meta-analysis of prevalence. J Epidemiol Community Health 2013;**67**(11):974-8.

16. Cochran GW. The Combination of Estimates from Different Experiments. Biometrics 1954;**10**(1):101-29.

17. Higgins JP, Thompson SG. Quantifying heterogeneity in a meta-analysis. Statistics in medicine 2002;**21**(11):1539-58.

18. Viera AJ, Garrett JM. Understanding interobserver agreement: the kappa statistic. Fam Med 2005;**37**(5):360-3.

19. Egger M, Davey Smith G, Schneider M, et al. Bias in meta-analysis detected by a simple, graphical test. BMJ 1997;**315**(7109):629-34.

20. Duval S, Tweedie R. Trim and fill: A simple funnel-plot-based method of testing and adjusting for publication bias in meta-analysis. Biometrics 2000;**56**(2):455-63.

21. Knobloch K, Yoon U, Vogt PM. Preferred reporting items for systematic reviews and meta-analyses (PRISMA) statement and publication bias. Journal of cranio-maxillo-facial surgery : official publication of the European Association for Cranio-Maxillo-Facial Surgery 2011;**39**(2):91-2.

22. Moher D, Shamseer L, Clarke M, et al. Preferred reporting items for systematic review and meta-analysis protocols (PRISMA-P) 2015 statement. Systematic reviews 2015;**4**:1.

For peer review only

For peer review only - http://bmjopen.bmj.com/site/about/guidelines.xhtml

Table 1. Search strategy in PubMed

Search	Search terms
#1	"HIV" OR "AIDS" OR "antiretroviral therapy" OR "antiretroviral treatment" OR "highly active antiretroviral therapy" OR "highly active ART"
#2	"Hypertension" OR "high blood pressure" OR "systolic hypertension" OR "diastolic hypertension" OR "anti-hypertensive" OR "blood pressure" OR "hypertensive"
#3	#1 AND #2
#4	#3 Limits: from 1996/01/01 to 2017/02/28

For peer review only

Table 2. Risk of bias assessment tool

**Risk of Bias Item***
External Validity
1. Was the study target population a close representation of the HIV population in relation to relevant variables?
2. Was the sampling frame a true or close representation of the target population?
3. Was some form of random selection used to select the sample, OR, was a census undertaken?
4. Was the likelihood of non-participation bias minimal?
Internal Validity
5. Were data collected directly from the subjects (as opposed to medical records)?
6. Were acceptable case definition of hypertension used?
7. Was a reliable and accepted diagnosis method for hypertension utilized?
8. Was the same mode of data collection used for all subjects?
9. Was the length of the shortest prevalence period for the parameter of interest appropriate?
10. Were the numerator(s) and denominator(s) for the calculation of the prevalence/incidence of hypertension appropriate?

*Adapted from the Risk of Bias Tool for Prevalence Studies developed by Hoy et al

For peer review only

For peer review only - http://bmjopen.bmj.com/site/about/guidelines.xhtml

PRISMA-P (Preferred Reporting Items for Systematic review and Meta-Analysis Protocols) 2015 checklist: recommended items to address in a systematic review protocol

Section and topic	Item No	Checklist item	
**ADMINISTRATIVE INFORMATION**			
Title:			
Identification	1a	Identify the report as a protocol of a systematic review	1
Update	1b	If the protocol is for an update of a previous systematic review, identify as such	-
Registration	2	If registered, provide the name of the registry (such as PROSPERO) and registration number	3
Authors:			
Contact	3a	Provide name, institutional affiliation, e-mail address of all protocol authors; provide physical mailing address of corresponding author	1
Contributions	3b	Describe contributions of protocol authors and identify the guarantor of the review	13
Amendments	4	If the protocol represents an amendment of a previously completed or published protocol, identify as such and list changes; otherwise, state plan for documenting important protocol amendments	-
Support:			
Sources	5a	Indicate sources of financial or other support for the review	13
Sponsor	5b	Provide name for the review funder and/or sponsor	-
Role of sponsor or funder	5c	Describe roles of funder(s), sponsor(s), and/or institution(s), if any, in developing the protocol	-
**INTRODUCTION**			
Rationale	6	Describe the rationale for the review in the context of what is already known	5
Objectives	7	Provide an explicit statement of the question(s) the review will address with reference to participants, interventions, comparators, and outcomes (PICO)	6
**METHODS**			
Eligibility criteria	8	Specify the study characteristics (such as PICO, study design, setting, time frame) and report characteristics (such as years considered, language, publication status) to be used as criteria for eligibility for the review	6-7
Information sources	9	Describe all intended information sources (such as electronic databases, contact with study authors, trial registers or other grey literature sources) with planned dates of coverage	7-8
Search strategy	10	Present draft of search strategy to be used for at least one electronic database, including planned limits, such that it could be repeated	7-8
Study records:			
Data management	11a	Describe the mechanism(s) that will be used to manage records and data throughout the review	8-9
Selection	11b	State the process that will be used for selecting studies (such as two independent reviewers) through each phase of the review	7

process		(that is, screening, eligibility and inclusion in meta-analysis)	
Data collection process	11c	Describe planned method of extracting data from reports (such as piloting forms, done independently, in duplicate), any processes for obtaining and confirming data from investigators	8-9
Data items	12	List and define all variables for which data will be sought (such as PICO items, funding sources), any pre-planned data assumptions and simplifications	8-9
Outcomes and prioritization	13	List and define all outcomes for which data will be sought, including prioritization of main and additional outcomes, with rationale	8-9
Risk of bias in individual studies	14	Describe anticipated methods for assessing risk of bias of individual studies, including whether this will be done at the outcome or study level, or both; state how this information will be used in data synthesis	10
Data synthesis	15a	Describe criteria under which study data will be quantitatively synthesised	10-11
	15b	If data are appropriate for quantitative synthesis, describe planned summary measures, methods of handling data and methods of combining data from studies, including any planned exploration of consistency (such as I^2, Kendall's τ)	10-11
	15c	Describe any proposed additional analyses (such as sensitivity or subgroup analyses, meta-regression)	10-11
	15d	If quantitative synthesis is not appropriate, describe the type of summary planned	10-11
Meta-bias(es)	16	Specify any planned assessment of meta-bias(es) (such as publication bias across studies, selective reporting within studies)	10-11
Confidence in cumulative evidence	17	Describe how the strength of the body of evidence will be assessed (such as GRADE)	10-11

For peer review only

BMJ Open

Prevalence and incidence of hypertension in the global HIV-infected population: a systematic review and meta-analysis protocol

Journal:	*BMJ Open*
Manuscript ID	bmjopen-2017-016531.R2
Article Type:	Protocol
Date Submitted by the Author:	08-Aug-2017
Complete List of Authors:	Bigna, Jean Joel; Centre Pasteur of Cameroon, Department of Epidemiology and Public Health; University of Paris Sud XI, Faculty of Medicine<br>Tankeu, Aurel; University of Yaoundé 1, Department of Internal Medicine and Specialties, Faculty of Medicine and Biomedical Sciences<br>Kaze, Arnaud; Harvard Medical School, Brigham and Women's Hospital<br>Noubiap, Jean Jacques; Groote Schuur Hospital and University of Cape Town, Department of Medicine<br>Nansseu, Jobert Richie; University of Yaoundé 1, Department of Public Health, Faculty of Medicine and Biomedical Sciences; Ministry of Public Health, Department of Disease, Epidemics and Pandemics Control
**Primary Subject Heading**:	Cardiovascular medicine
Secondary Subject Heading:	Infectious diseases, Epidemiology, HIV/AIDS, Public health
Keywords:	HIV & AIDS < INFECTIOUS DISEASES, Hypertension < CARDIOLOGY, Epidemiology < INFECTIOUS DISEASES

SCHOLARONE™
Manuscripts

Prevalence and incidence of hypertension in the global HIV-infected population: a systematic review and meta-analysis protocol

Jean Joel Bigna[1, 2], Aurel T. Tankeu[3], Arnaud D. Kaze[4], Jean Jacques Noubiap[5], Jobert Richie Nansseu[6, 7]

[1-] Department of Epidemiology and Public Health, Centre Pasteur of Cameroon, Yaoundé, Cameroon

[2-] Faculty of Medicine, University of Paris Sud XI, Le Kremlin-Bicêtre, France

[3-] Department of Internal Medicine and Specialties, Faculty of Medicine and Biomedical Sciences, University of Yaoundé 1, Yaoundé, Cameroon

[4-] Brigham and Women's Hospital, Harvard Medical School, Boston, MA, USA

[5-] Department of Medicine, Groote Schuur Hospital and University of Cape Town, Cape Town, South Africa

[6-] Department of Public Health, Faculty of Medicine and Biomedical Sciences, University of Yaoundé 1, Yaoundé, Cameroon

[7-] Department of Disease, Epidemics and Pandemics Control, Ministry of Public Health, Yaoundé, Cameroon

* Correspondence to Dr Jean Joel Bigna, Department of Epidemiology and Public Health, Centre Pasteur of Cameroon, Yaoundé, Cameroon. Email: bignarimjj@yahoo.fr

E-mail addresses: JJB: bignarimjj@yahoo.fr; ATT: aurelnet2014@yahoo.com; ADK: kazearnaud@yahoo.fr; JJN: noubiapjj@yahoo.fr; JRN: jobertrichie_nansseu@yahoo.fr

Keywords: hypertension, HIV, AIDS, epidemiology

For peer review only

For peer review only - http://bmjopen.bmj.com/site/about/guidelines.xhtml

Abstract

Introduction

Hypertension representing one of the most frequent cardiovascular risk factor, is thought to increase in individuals living with HIV as well as in general population but summarized global data on the topic are scare. We conduct this systematic review and meta-analysis to assess the prevalence/incidence of hypertension in the global HIV-infected population.

Methods and analysis

This review will include observational studies conducted among HIV infected people, which reported prevalence/incidence of hypertension or enough data for its appraisal. We will consider published and unpublished studies from January 1, 2007 to May 31, 2017. Relevant records will be searched using PubMed/Medline, Global Index Medicus, Web of Science, and EMBASE. Reference lists of eligible papers and relevant review articles will be screened. Two investigators will independently screen, select studies, and extract data, with discrepancies resolved by consensus or by arbitration of a third investigator. Methodological quality of the included studies will be assessed using the scale developed by Hoy and colleagues. Funnel-plots and Egger's test will be used to determine publication bias. The study-specific estimates will be pooled through a random-effects meta-analysis model to obtain an overall summary estimate. To keep the effect of studies with extremely small or extremely large estimates on the overall estimate to a minimum, the variance of the study-specific prevalence/incidence will be stabilized with the Freeman-Tukey single arc-sine transformation. The heterogeneity will be evaluated by the χ^2 test on Cochrane's Q statistic. Results will be presented by geographic region, income and antiretroviral therapy status.

Ethic and Dissemination

For peer review only

For peer review only - http://bmjopen.bmj.com/site/about/guidelines.xhtml

This study is based on published data; therefore, ethical approval is not a requirement. The final report of this study in the form of a scientific paper will be published in peer-reviewed journals.

Review registration number

PROSPERO, CRD42016051684.

For peer review only - http://bmjopen.bmj.com/site/about/guidelines.xhtml

Strengths and limitations of this study

- To the best of our knowledge, this will be the first systematic review and meta-analysis which aims to estimate the global prevalence and incidence of hypertension in HIV infected people.
- We will use rigorous methodological and statistical procedures to derive accurate estimates.
- This review would be limited by the impossibility to disaggregate data between patients on antiretroviral therapy and those naïve of antiretroviral therapy.
- Another possible limitation could be the predominance of hospital-based studies limiting the generalizability of findings.

For peer review only

For peer review only - http://bmjopen.bmj.com/site/about/guidelines.xhtml

Introduction

The HIV pandemic is to-date one of the major global public health concern. Globally, there were 38.7 million people living with HIV in 2015 causing 35 million of deaths since the beginning of the epidemic.[1] Since the expanding use of highly active antiretroviral therapy (ART) in 1996, the natural history of HIV disease has been strikingly changed. This led to the increase of life expectancy in people living with HIV[1] and acceleration of epidemiological transition, from domination of the infectious diseases to non-communicable diseases.[2] The leading group of non-communicable diseases, cardiovascular diseases (CVDs), accounts for approximately 17 million deaths a year, nearly one third of the total worldwide. Of these, complications of hypertension account for more than half of deaths worldwide every year.[3 4]

Although the 38.7 million people living with HIV can now live longer because of ART, the risk of CVD including hypertension increases with aging and other factors such as diet, physical inactivity, smoking, and hyperuricemia.[5-8] This is due to both HIV and ART. Indeed, several HIV related factors have been mentioned to cause vascular dysfunction by HIV itself including endothelial dysfunction, endothelial cells activation through viral proteins action, activation of macrophages responsible of accelerated atheroma formation, HIV-associated lipid disorders, proinflammatory and prothrombotic state, and direct HIV infection of endothelial cells and vascular smooth muscle cells.[9 10] In addition to HIV itself, ART have been incriminated to increase the oxidative stress in endothelial cells, to favor adhesion of mononuclear on vascular endothelium and insulin resistance, to increase accumulation of fatty acids and lipids on vessel wall, and to favor persistent immunity activity and inflammation.[10] Autonomic neuropathy and vasculitis were also evocated as potential mechanism for the occurrence of hypertension in HIV people by a secondary pathogenic pathway.[9] Given the recent World Health Organization (WHO) recommendations for initiating ART in all HIV-infected people regardless of CD4 count;[11 12] people living with

For peer review only - http://bmjopen.bmj.com/site/about/guidelines.xhtml

HIV will face the double burden of HIV infection and HIV and ART induced CVDs including hypertension. One can also wonder whether countries with weak health system are ready to face these challenges.[7 12] This is why it is important to know what is the burden of CVDs in people living with HIV?

To date, although there are several original studies that report the prevalence and incidence of hypertension in people living with HIV, there is no systematic global evidence to the best of our knowledge. This review will be an opportunity to discuss epidemiological data across different WHO regions, income level, and socio-demographic profiles. We therefore plan to conduct this systematic review and meta-analysis to address this gap.

Review question

What is the prevalence and incidence of hypertension in the global HIV-infected population as documented in studies reported between January 1st, 2006 and July 31st, 2017?

Objectives

To answer this review question, a systematic review and meta-analysis will be conducted. The objectives of which are to determine:

1) The prevalence and incidence of hypertension in the global HIV-infected ART-naive population,
2) The prevalence and incidence of hypertension in the global HIV-infected ART-experienced population.

Methods and analysis

For peer review only - http://bmjopen.bmj.com/site/about/guidelines.xhtml

Design

Centre for Reviews and Dissemination guidelines will be used for the methodology of this review.[13] This review is registered in the PROSPERO International Prospective Register of systematic reviews, registration number CRD42016051684.

Criteria for considering studies for this review

Inclusion criteria

1) Types of studies: observational studies (cross-sectional, case-control, or cohort studies).
2) Types of participants: HIV-infected ART-naïve people and HIV-infected ART-experienced people.
3) Type of outcome: hypertension defined as office blood pressure ≥ 140/90 mmHg and/or treatment with anti-hypertensive medications.
4) Outcome measurement: prevalence/incidence or having enough data to compute these estimates.
5) Type of publication: published and unpublished studies reported from January 1, 2007 to May 31, 2017.

Exclusion criteria

1) Types of studies: case series, reviews, policy reports, commentaries and editorials.
2) Types of outcomes: studies on non-systemic and non-essential hypertension (intracranial hypertension, pulmonary hypertension).

For peer review only - http://bmjopen.bmj.com/site/about/guidelines.xhtml

3) Types of participants: studies in subgroups of participants selected on the basis of the presence of hypertension. Studies in which it will not be possible to differentiate HIV-infected ART-naive people and HIV-infected ART-experienced people. Studies conducted or in which it will not be possible to differentiate data of adults with those of children, adolescents, or pregnant women.

4) Outcome measurement: studies lacking primary data and/or explicit method description.

5) Duplicate reports: the most comprehensive and up-to-date version will be considered for this review.

Search strategy for identifying relevant studies

Bibliographic database search

A comprehensive and exhaustive search of PubMed/Medline, Global Index Medicus, Web of Science, and EMBASE will be performed to identify all relevant articles published on systemic hypertension among HIV-infected people between January 1, 2007 and May 31, 2017. A search strategy based on the combination of relevant terms will be conceived and applied. The following terms will be used for hypertension: “hypertension”, “high blood pressure”, “systolic hypertension”, “diastolic hypertension”, “blood pressure”, “anti-hypertensive”, and “hypertensive”. For HIV, we will use the terms “HIV”, “AIDS” and “antiretroviral”. The main search strategy conducted in PubMed/Medline is shown in Table 1. This search strategy will be adapted for searching other database.

Searching other sources

For peer review only - http://bmjopen.bmj.com/site/about/guidelines.xhtml

A manual search which consists in scanning the reference lists of eligible papers and other relevant review articles will be conducted.

Selection of studies for inclusion in the review

Two investigators will independently identify articles and sequentially screen their titles and abstracts for eligibility. Full-texts of articles deemed potentially eligible will be retrieved. Further, these investigators will independently assess the full-text of each study for eligibility, and consensually retain studies to be included. Disagreements when existing will be solved by a third investigator. A screening guide will be used to ensure that the selection criteria are reliably applied by all investigators. Selection of studies will be managed using EndNote X7.

Data extraction and management

Two investigators will independently extract data pertaining to:

1) Author details: name of first author and publication year.

2) Study characteristics: country, WHO region (African Region, Regions of Americas, South-East Asia Region, European Region, Eastern Mediterranean Region, and Western Pacific Region), World Bank classification by income level at the time of data collection (low-income, lower-middle-income, upper-middle income, high-income countries), study design (cross sectional, case-control, cohort), setting (urban and/or rural), sampling method (random vs non-random), data collection period, response rate, timing of data collection (prospectively vs retrospectively), and methodological quality of the study.

For peer review only - http://bmjopen.bmj.com/site/about/guidelines.xhtml

3) Participants' characteristics: selection criteria, age (mean, median, range), gender (proportion of male), HIV related data (time since diagnosis, severity of the disease, ART regimens and duration of treatment).

4) Hypertension characteristics: diagnostic criteria used, prevalence, number of participants tested and diagnosed with hypertension overall and by subgroup of interest like level of urbanization, gender and ART regimens and status.

Where only primary data (sample size and number of outcomes) will be provided, these data will be used to calculate the prevalence or incidence estimates. Data will be extracted using a preconceived and standardized data abstraction form. Disagreements between investigators will be reconciled through discussion and consensus, or arbitration by a third investigator whenever necessary. In case of multinational studies, the results will be separated to show the estimate within individual countries. When it will not be possible to disaggregate the data by country, the study will be presented as one and the countries in which the study was done will be shown.

Appraisal of the methodological quality of included studies and risk of bias

Included studies will be evaluated for methodological quality in terms of internal validity, external validity, response rate, and generalizability of study results. We will adapt the 10-item rating scale developed Hoy et al (Table 2).[14] Each item will be assigned a score of 1 (Yes) or 0 (No), and score will be summed across items to generate an overall quality score. The total score will range from 0 to 10 with the overall score categorized as follows: 8-10: "low risk of bias", 5-7: "moderate risk", and 0-4: "high risk". We intend to present risk of bias and quality scores in a table.

For peer review only - http://bmjopen.bmj.com/site/about/guidelines.xhtml

Data synthesis including assessment of heterogeneity

Data will be analyzed using Stata software (Stata Corp V.14, Texas, USA). Unadjusted prevalence/incidence and standard errors of hypertension will be recalculated based on the information of crude numerators and denominators provided by individual studies. To keep the effect of studies with extremely small or extremely large prevalence estimates on the overall estimate to a minimum, the variance of the study-specific prevalence/incidence will be stabilized with the Freeman-Tukey single arc-sine transformation before pooling the data with the random-effects meta-analysis model.[15] Heterogeneity will be evaluated by the Chi-squared test on Cochrane's Q statistic,[16] which will be quantified by I-squared values, assuming that I-squared values of 25%, 50% and 75% being representative of low, medium and high heterogeneity respectively.[17] When substantial heterogeneity will be detected, we will perform a subgroup and metaregression analyses to investigate the possible sources of heterogeneity using the following grouping variables: age group, sex, study setting (rural vs urban), geographical area, country income level, sampling method, timing of data collection, ART regimens and status, hypertension diagnosis criteria, and study methodological quality. We will assess inter-rater agreement between investigators for study inclusion, data extraction, and methodological quality assessment using Kappa Cohen's coefficient.[18] If the included studies differ significantly in design, settings, outcome measures or otherwise, a narrative format will be used to summarize data of studies.

Assessment of reporting biases

Symmetry of funnel plots and Egger's test will be done to assess the presence of publication and selective reporting bias.[19] A p-value < 0.10 will be considered indicative of statistically significant publication bias. In addition, we will perform a trim-and-fill adjusted analysis.

For peer review only - http://bmjopen.bmj.com/site/about/guidelines.xhtml

This help to perform the Duval and Tweedie nonparametric "trim and fill" method of accounting for publication bias in meta-analysis and estimates the number and outcomes of missing studies, and adjusts the meta-analysis to incorporate the theoretical missing studies.[20]

Reporting of this review

Results will be presented by geographic region, country income level and antiretroviral therapy status. The guidelines for meta-analyses and systematic reviews of Preferred Reporting Items for Systematic Review and Meta-Analysis (PRISMA) will serve as the template for reporting the present review.[21] For the present protocol, the PRISMA for Protocol was used for the reporting (see online supplementary appendix S1).[22]

Potential amendments

We do not intend to make any amendments to the protocol, to avoid the possibility of outcome reporting bias. However, any amendments during review process will be reported transparently.

Conclusions

Reduction of mortality and morbidity gained in HIV infected individuals with the widespread use of HAART has been accompanied by an increasing prevalence of CVD in this population. [5-7] Hypertension which as far the most frequent and worrying cardiovascular risk factor is increasing among HIV infected patients as in general population. In this context, we

For peer review only - http://bmjopen.bmj.com/site/about/guidelines.xhtml

plan to conduct this review with the aim of estimating the burden of this condition in this population. We hope that this review will serve to draw attention and raise awareness on this growing concern.

Ethics and dissemination

The current study is based on published data and as such, ethical approval is not a requirement. The final report of the systematic review in the form of a scientific paper will be published in peer-reviewed journals. Findings will further be presented at conferences and submitted to relevant health authorities. We also plan to update the review in the future to monitor changes and guide health service and policy solutions.

Competing interests

None.

Contributors

Conception and design of the protocol: ATT and JJRB. First draft: ATT and JJRB. Critical revision of the manuscript for methodological and intellectual content: JRNN, ADK, and JJNN. Guarantor of the review: JJRB. All authors approved the final version of the submitted manuscript.

Funding

This research received no specific grant from any funding agency in the public, commercial or not-for-profit sectors.

Data sharing statement

For peer review only - http://bmjopen.bmj.com/site/about/guidelines.xhtml

No additional data are available.

References

1. UNAIDS. Fact sheet 2016 Global statistics-2015. Secondary Fact sheet 2016 Global statistics-2015 2016 2016. http://www.unaids.org/en/resources/fact-sheet.

2. Kuate Defo B. Demographic, epidemiological, and health transitions: are they relevant to population health patterns in Africa? Global health action 2014;**7**:22443.

3. World Health Organization. A global brief on hypertension. Secondary A global brief on hypertension 2013. http://apps.who.int/iris/bitstream/10665/79059/1/WHO_DCO_WHD_2013.2_eng.pd.

4. Lim SS, Vos T, Flaxman AD, et al. A comparative risk assessment of burden of disease and injury attributable to 67 risk factors and risk factor clusters in 21 regions, 1990-2010: a systematic analysis for the Global Burden of Disease Study 2010. Lancet 2012;**380**(9859):2224-60.

5. Dillon DG, et al. Association of HIV and ART with cardiometabolic traits in sub-Saharan Africa: a systematic review and meta-analysis. Int J Epidemiol 2016.

6. Nduka CU, Stranges S, Sarki AM, et al. Evidence of increased blood pressure and hypertension risk among people living with HIV on antiretroviral therapy: a systematic review with meta-analysis. Journal of human hypertension 2016;**30**(6):355-62.

7. Nansseu JR, Bigna JJ. Antiretroviral therapy related adverse effects: Can sub-Saharan Africa cope with the new "test and treat" policy of the World Health Organization? Infectious diseases of poverty 2017;**6**(1):24.

8. Ances BM, Bhatt A, Vaida F, et al. Role of metabolic syndrome components in human immunodeficiency virus-associated stroke. Journal of neurovirology 2009;**15**(3):249-56.

9. Calo LA, Caielli P, Maiolino G, et al. Arterial hypertension and cardiovascular risk in HIV-infected patients. Journal of cardiovascular medicine (Hagerstown, Md) 2013;**14**(8):553-8.

10. Dube MP, Lipshultz SE, Fichtenbaum CJ, et al. Effects of HIV infection and antiretroviral therapy on the heart and vasculature. Circulation 2008;**118**(2):e36-40.

11. World health Organization. Consolidated guidelines on the use of antiretroviral drugs for treating and preventing HIV infection - Recommendations for a public health approach - Second edition. Secondary Consolidated guidelines on the use of antiretroviral drugs for treating and preventing HIV infection - Recommendations for a public health approach - Second edition June 2016 2016. http://www.who.int/hiv/pub/arv/arv-2016/en/.

For peer review only - http://bmjopen.bmj.com/site/about/guidelines.xhtml

12. Bigna JJ, Plottel CS, Koulla-Shiro S. Challenges in initiating antiretroviral therapy for all HIV-infected people regardless of CD4 cell count. Infectious diseases of poverty 2016;**5**:85.

13. Centers for Reviews and Dissemination. CRD's guidance for undertaking reviews in healthcare: Centers for Reviews and Dissemination, 2009.

14. Hoy D, Brooks P, Woolf A, et al. Assessing risk of bias in prevalence studies: modification of an existing tool and evidence of interrater agreement. Journal of clinical epidemiology 2012;**65**(9):934-9.

15. Barendregt JJ, Doi SA, Lee YY, et al. Meta-analysis of prevalence. J Epidemiol Community Health 2013;**67**(11):974-8.

16. Cochran GW. The Combination of Estimates from Different Experiments. Biometrics 1954;**10**(1):101-29.

17. Higgins JP, Thompson SG. Quantifying heterogeneity in a meta-analysis. Statistics in medicine 2002;**21**(11):1539-58.

18. Viera AJ, Garrett JM. Understanding interobserver agreement: the kappa statistic. Fam Med 2005;**37**(5):360-3.

19. Egger M, Davey Smith G, Schneider M, et al. Bias in meta-analysis detected by a simple, graphical test. BMJ 1997;**315**(7109):629-34.

20. Duval S, Tweedie R. Trim and fill: A simple funnel-plot-based method of testing and adjusting for publication bias in meta-analysis. Biometrics 2000;**56**(2):455-63.

21. Knobloch K, Yoon U, Vogt PM. Preferred reporting items for systematic reviews and meta-analyses (PRISMA) statement and publication bias. Journal of cranio-maxillo-facial surgery : official publication of the European Association for Cranio-Maxillo-Facial Surgery 2011;**39**(2):91-2.

22. Moher D, Shamseer L, Clarke M, et al. Preferred reporting items for systematic review and meta-analysis protocols (PRISMA-P) 2015 statement. Systematic reviews 2015;**4**:1.

For peer review only - http://bmjopen.bmj.com/site/about/guidelines.xhtml

326 **Table 1. Search strategy in PubMed**

Search	Search terms
#1	"HIV" OR "AIDS" OR "antiretroviral therapy" OR "antiretroviral treatment" OR "highly active antiretroviral therapy" OR "highly active ART"
#2	"Hypertension" OR "high blood pressure" OR "systolic hypertension" OR "diastolic hypertension" OR "anti-hypertensive" OR "blood pressure" OR "hypertensive"
#3	#1 AND #2
#4	#3 Limits: from 1996/01/01 to 2017/02/28

327

328

For peer review only

For peer review only - http://bmjopen.bmj.com/site/about/guidelines.xhtml

329 **Table 2. Risk of bias assessment tool**

**Risk of Bias Item***
External Validity
1. Was the study target population a close representation of the HIV population in relation to relevant variables?
2. Was the sampling frame a true or close representation of the target population?
3. Was some form of random selection used to select the sample, OR, was a census undertaken?
4. Was the likelihood of non-participation bias minimal?
Internal Validity
5. Were data collected directly from the subjects (as opposed to medical records)?
6. Were acceptable case definition of hypertension used?
7. Was a reliable and accepted diagnosis method for hypertension utilized?
8. Was the same mode of data collection used for all subjects?
9. Was the length of the shortest prevalence period for the parameter of interest appropriate?
10. Were the numerator(s) and denominator(s) for the calculation of the prevalence/incidence of hypertension appropriate?

330 *Adapted from the Risk of Bias Tool for Prevalence Studies developed by Hoy et al

331

332

For peer review only

For peer review only - http://bmjopen.bmj.com/site/about/guidelines.xhtml

PRISMA-P (Preferred Reporting Items for Systematic review and Meta-Analysis Protocols) 2015 checklist: recommended items to address in a systematic review protocol

Section and topic	Item No	Checklist item	
**ADMINISTRATIVE INFORMATION**			
Title:			
Identification	1a	Identify the report as a protocol of a systematic review	1
Update	1b	If the protocol is for an update of a previous systematic review, identify as such	-
Registration	2	If registered, provide the name of the registry (such as PROSPERO) and registration number	3
Authors:			
Contact	3a	Provide name, institutional affiliation, e-mail address of all protocol authors; provide physical mailing address of corresponding author	1
Contributions	3b	Describe contributions of protocol authors and identify the guarantor of the review	13
Amendments	4	If the protocol represents an amendment of a previously completed or published protocol, identify as such and list changes; otherwise, state plan for documenting important protocol amendments	-
Support:			
Sources	5a	Indicate sources of financial or other support for the review	13
Sponsor	5b	Provide name for the review funder and/or sponsor	-
Role of sponsor or funder	5c	Describe roles of funder(s), sponsor(s), and/or institution(s), if any, in developing the protocol	-
**INTRODUCTION**			
Rationale	6	Describe the rationale for the review in the context of what is already known	5
Objectives	7	Provide an explicit statement of the question(s) the review will address with reference to participants, interventions, comparators, and outcomes (PICO)	6
**METHODS**			
Eligibility criteria	8	Specify the study characteristics (such as PICO, study design, setting, time frame) and report characteristics (such as years considered, language, publication status) to be used as criteria for eligibility for the review	6-7
Information sources	9	Describe all intended information sources (such as electronic databases, contact with study authors, trial registers or other grey literature sources) with planned dates of coverage	7-8
Search strategy	10	Present draft of search strategy to be used for at least one electronic database, including planned limits, such that it could be repeated	7-8
Study records:			
Data management	11a	Describe the mechanism(s) that will be used to manage records and data throughout the review	8-9
Selection	11b	State the process that will be used for selecting studies (such as two independent reviewers) through each phase of the review	7

process		(that is, screening, eligibility and inclusion in meta-analysis)	
Data collection process	11c	Describe planned method of extracting data from reports (such as piloting forms, done independently, in duplicate), any processes for obtaining and confirming data from investigators	8-9
Data items	12	List and define all variables for which data will be sought (such as PICO items, funding sources), any pre-planned data assumptions and simplifications	8-9
Outcomes and prioritization	13	List and define all outcomes for which data will be sought, including prioritization of main and additional outcomes, with rationale	8-9
Risk of bias in individual studies	14	Describe anticipated methods for assessing risk of bias of individual studies, including whether this will be done at the outcome or study level, or both; state how this information will be used in data synthesis	10
Data synthesis	15a	Describe criteria under which study data will be quantitatively synthesised	10-11
	15b	If data are appropriate for quantitative synthesis, describe planned summary measures, methods of handling data and methods of combining data from studies, including any planned exploration of consistency (such as I^2, Kendall's τ)	10-11
	15c	Describe any proposed additional analyses (such as sensitivity or subgroup analyses, meta-regression)	10-11
	15d	If quantitative synthesis is not appropriate, describe the type of summary planned	10-11
Meta-bias(es)	16	Specify any planned assessment of meta-bias(es) (such as publication bias across studies, selective reporting within studies)	10-11
Confidence in cumulative evidence	17	Describe how the strength of the body of evidence will be assessed (such as GRADE)	10-11

For peer review only
